# Students Eat Less Meat After Studying Meat Ethics

**DOI:** 10.1007/s13164-021-00583-0

**Published:** 2021-11-06

**Authors:** Eric Schwitzgebel, Bradford Cokelet, Peter Singer

**Affiliations:** 1 Department of Philosophy, University of California at Riverside, Riverside, CA 92521-0201 USA; 2 University of Kansas, Lawrence, KS USA; 3 Princeton University, Princeton, NJ USA

## Abstract

In the first controlled, non-self-report studies to show an influence of university-level ethical instruction on everyday behavior, Schwitzgebel et al. (2020) and Jalil et al. (2020) found that students purchase less meat after exposure to material on the ethics of eating meat. We sought to extend and conceptually replicate this research. Seven hundred thirty students in three large philosophy classes read James Rachels’ (2004) “Basic Argument for Vegetarianism”, followed by 50-min small-group discussions. Half also viewed a vegetarianism advocacy video containing factory farm footage. A few days after instruction, 54% of students agreed that “eating the meat of factory farmed animals is unethical”, compared to 37% before instruction, with no difference between the film and non-film conditions. Also, 39% of students anonymously pledged to avoid eating factory farmed meat for 24 h, again with no statistically detectable difference between conditions. Finally, we obtained 2828 campus food purchase receipts for 113 of the enrolled students who used their Student ID cards for purchases on campus, which we compared with 5033 purchases from a group of 226 students who did not receive the instruction. Meat purchases remained constant in the comparison group and declined among the students exposed to the material, falling from 30% to 23% of purchases overall and from 51% to 42% of purchases of $4.99 or more, with the effect possibly larger in the film condition.

**Supplementary Information:**

The online version contains supplementary material available at 10.1007/s13164-021-00583-0.

Does philosophical instruction have any influence on students’ real world behavior outside of the university classroom? And if it does have an influence, what are the mechanisms of that influence? These questions should interest anyone concerned about the practical value of academic philosophy. Most academic philosophers spend a substantial proportion of their professional time in instructional activities. If those instructional activities influence students’ behavior not only inside the classroom (e.g., their test taking) but also outside the classroom (e.g., their life choices), that is potentially an important aspect of the discipline’s practical value. Philosophy classroom instruction might not only enable students to perform well on examinations about what Plato said; it might also alter their lives.

Some, but not all, instructors, administrators, and students desire such an impact from philosophy classes. The three authors of this article, for example, all hope that our students will think and act somewhat differently and better as a result of our courses. Historically, philosophy requirements, especially applied ethics classes, are often justified by the expectation that students will carry the lessons beyond the classroom (Abend 2014). Indeed, a majority of business ethics students describe the acquisition of “practical knowledge that will help me be a more ethical business leader in the future” as extremely important to them – as important as or more important than grades and satisfying degree requirements (Schwitzgebel and Strohminger 2020). Relatedly, Martha Nussbaum (1997) has argued that a broad humanities education, including philosophy, is culturally important on the grounds that it produces better “world citizens”, improving students’ contributions to political and other decisions.

There is, however, little systematic evidence that philosophy classroom instruction has any influence on students’ behavior outside the classroom. In general, even for more straightforwardly applied disciplines such as basic physics and mathematics, students often fail to transfer knowledge acquired in the classroom to analogous life situations (Barnett and Ceci 2002; Perkins and Salomon 2012; Richland et al. 2012; Caplan 2018). Transference might even be less for philosophy, since the topics of philosophy often have little obvious direct bearing on students’ lives (in what concrete ways ought one act differently in the world after having read Kant’s *Critique of Pure Reason?*) and also since philosophers often teach opposing sides of an argument (e.g., utilitarianism, pro and con) leaving students to decide for themselves which of the alternatives if any is correct.

Furthermore, in a series of studies of the moral behavior of professional ethicists, Eric Schwitzgebel and Joshua Rust found that ethicists did not behave any morally better than other professors with whom they were compared, across a wide range of measures, including charitable donation, organ and blood donation, answering student emails, political participation, staying in touch with their mothers, discourteous behavior at conferences, stealing library books, littering, joining the Nazi party in 1930s Germany, and overall moral evaluation by peers (Schwitzgebel and Rust 2009, 2014, 2016; Schwitzgebel 2019b; see also Schönegger and Wagner 2019). In addition, studies of the instructional effectiveness of business ethics and medical ethics classes on indirect measures of real-world behavior (e.g., laboratory behavior or self-reported intentions) tend to find modest to non-existent effects, despite methodological flaws that would plausibly lead to the overreporting of positive findings (Schwitzgebel 2013). These empirical findings invite, but do not compel, the idea that ethics instruction has no measurable impact on student behavior. If professional ethicists’ long exposure to the material of ethics has no detectable influence on their behavior, why expect that shorter-term exposure to similar material would have any effect on students?

Summing up, armchair reflection about the behavioral impact of ethics education pulls in two directions. Based on general plausibility, one might tend to assume, with Abend (2014) and Nussbaum (1997), that philosophical instruction will influence students’ behavior outside the classroom. But based on other plausibility considerations, and the indirect empirical evidence described in the previous two paragraphs, one might assume instead that students will master only what they need to pass tests, proceeding with their daily lives approximately unchanged.

The question has almost never been addressed with empirical rigor. It is difficult to address empirically due to both logistical and measurement challenges. The measurement challenge is this: Researchers need a feasible measure of real-world moral behavior that they anticipate would be substantially affected by philosophical instruction. However, a practical measure of this sort may be difficult to conceive. We have, for example, no meter by which to straightforwardly measure former students’ thoughtfulness, their overall conformity to consequentialist (or deontological) ethical principles, or their excellence as world citizens. The logistical challenge is this: Whatever measure is devised, we ought realistically to expect only modest and noisy effects, given the generally modest transference of even straightforward skills outside the university classroom. Therefore, a good experiment will require high statistical power. It will require large samples of students assigned to at least two different instructional conditions, whose outside-the-classroom behavior can then later be measured. This necessitates a large, cooperative university setting and the coordination of people and resources far exceeding that involved in the typical study.

As far as we are aware, the only published experimental work that directly measures the real-world effects of university level philosophy instruction concerns the ethics of eating meat. Meat ethics is an attractive topic of study for four reasons. First, there is widespread consensus among philosophers writing on the topic that it is generally morally better for typical North Americans to reduce or avoid consuming factory farmed meat (Singer, 1975/2009; Regan 1983; Adams 1990/2015; DeGrazia 1996; Scruton 2004; Pollan 2006; Gruen 2012; Camosy 2013; Korsgaard 2018; Huemer 2019). This makes it likely that if there is any effect of instruction on behavior, it will be unidirectional, toward eating less meat, rather than bidirectional as it might be for a more controversial issue. Second, campus food purchase choices are a directly measurable real-world behavior without need of potentially problematic self-report, are large in number, and can be systematically linked with student identification numbers through campus vendors, making for an attractive and powerful dependent measure. Third, anecdotally, it is not uncommon for university students already to be considering meat elimination or meat reduction, and professors who teach meat ethics often report that after learning the material students sometimes express an interest in becoming vegetarian. This suggests possible receptivity to behavioral change on exposure to classroom instruction. Finally, the literature on knowledge transference outside of the classroom suggests that transfer is more likely when the connection between the in-classroom and out-of-classroom material is easy to detect and recall, as would appear to be the case for meat ethics.

Two published studies suggest an effect of classroom instruction on students’ meat purchasing behavior. Schwitzgebel et al. (2020) divided 1143 students in four large lower division philosophy classes at University of California, Riverside into two conditions. In one condition, students read James Rachels’ 10-page article “The Basic Argument for Vegetarianism” (Rachels 2004), which argues that people’s enjoyment of the taste of meat does not justify the enormous suffering that factory farmed animals endure. Students then discussed the argument’s merits in small (~20 person), 50 min discussion meetings. These discussions were led by teaching assistants (TAs) all of whom were practicing vegetarians. The TAs were encouraged to teach the material in their ordinary teaching style, and some revealed their personal opinions and behavior, though this was not systematically measured. Students were also provided with a link to an optional 11-min vegetarianism advocacy video, which 33% reported having watched and another 25% reported having started without finishing. The other half of students received comparable instruction on the ethics of charitable giving. Campus food purchase receipts were available for 495 of the 1143 students, comprising 13,642 purchases total. Students in the meat ethics condition purchased less meat at campus locations after instruction, declining from 28% of purchases overall containing meat before instruction to 25% after (p = .004), and declining from 52% to 45% among purchases of at least $4.99 (p = .001), with an estimated duration of at least several weeks. In the comparison group, purchase behavior did not change. In an anonymous questionnaire distributed a few days after instruction, students were substantially more likely to agree that “eating the meat of factory farmed animals is unethical” in the meat ethics condition than in the comparison condition (43% vs. 29% agreeing, p < .001).

Jalil et al. (2020) studied the on-campus purchase behavior of 215 students in ten economics classes at Occidental College. Half of the classes received about 55 min of instruction on the negative climate effects of meat production and the health benefits of a vegetarian diet (the treatment condition) and the other half received instruction on economic inequality (the control). Although these are economics rather than philosophy students, we include this study here because of its design similarity to Schwitzgebel et al. (2020) and because climate change is an ethical issue. Jalil, Tasoff, and Bustamante examined 49,289 on-campus lunch and dinner purchases by these 215 students over the course of an entire academic year, finding that, among students in the treatment condition, the percentage of meat purchases declined from about 58% to about 54% immediately after the intervention, then slowly returned to approximately pre-intervention levels by the end of the year. Students in the control condition showed a roughly constant rate of meat purchases, approximately 65–67% throughout the period. (Note that the control group purchased more meat than the treatment group even before the intervention, presumably due to chance imbalances in assignment.) Though not directly measuring behavior, Feltz & Feltz (2019) and Wright (2020) also found that exposure to material on meat ethics can lead some people to reduce their self-reported meat consumption.

Although the effect sizes reported in Schwitzgebel et al. (2020) and Jalil et al. (2020) are small – just a few percentage points – this is what would be theoretically predicted, given the literature on weak transference and the history of null results concerning the behavior of ethics professors. We would not expect the majority of students to dramatically change their daily behavior after brief exposure to philosophical instruction. However, even small reductions in meat consumption are substantial in terms of animal welfare: If everyone in the United States reduced their meat consumption by 3%, 256 million fewer land-based vertebrate animals would be reared and die as part of the meat industry annually – with 98% of them confined in factory farms (Anthis 2019; Humane Ventures 2021).

The present study aims to extend and conceptually replicate the results of the two studies just described. The value and importance of replication in the social science has been increasingly recognized in recent years (Open Science Collaboration 2015; Freese and Peterson 2017; Machery 2020). Replication is especially desirable for the studies in question, given the complexity of the datasets (inviting potential concerns about analytic choices and researcher degrees of freedom), the imperfect balancing and randomization (for example, the higher meat consumption rate in Jalil and colleagues’ control group even before intervention), the small effect sizes (small effects being potentially more explainable by small imperfections in design or analysis), the relatively small number of instructors (who might be unusually inspiring or uninspiring and thus not representative of a typical instructional situation), and the fact that neither study was fully preregistered.

We also sought to better explore the basis of behavioral change. Since Jalil et al. (2020) paired the material on climate change with the material on the health benefits of vegetarianism, it is unclear to what extent ethical considerations, as opposed to health considerations, drove the effect they found. Since Schwitzgebel et al. (2020) gave participants a link to an advocacy video containing factory farm footage, which about half of participants reported at least starting to watch, it is possible that the video drove the effect and the more traditional aspects of philosophy instruction – the required reading and classroom discussion – would have had no effect in isolation. Furthermore, since all of the TAs in the treatment condition in Schwitzgebel et al. (2020) were practicing vegetarians, it remains unclear to what extent students were influenced by TAs’ personal attitudes (either explicitly reported or implicitly conveyed), or a biased presentation, as opposed to the arguments and considerations in the assigned material as that material would be presented by instructors not personally committed to vegetarianism.

The present study builds upon the methods of Schwitzgebel et al. (2020). We address the concern about whether the exposure to the film was essential to the effect by dividing students into two groups, half of whom saw the film and the other half of whom did not, to better isolate the effect of the film versus other effects. We address the concern about the relevance of TAs’ personal attitudes by employing TAs with a variety of dietary habits, rather than only vegetarians, and examining whether the effect is also present among students with non-vegetarian TAs. We also sought to extend and improve the questionnaire method of Schwitzgebel et al. (2020) by including questionnaires both pre and post to better track attitude change, and improving the language of one of the questionnaire items. Finally, we added a new dependent measure, a “pledge opportunity”, an easy and possibly attractive chance to experiment with meat reduction by pledging not to consume factory farmed meat for the next 24 h.

We had two main hypotheses. First, students exposed to philosophical arguments for vegetarianism would purchase less meat from campus dining locations than a comparison group not exposed to the instruction, conceptually replicating earlier research. Second, this effect would be larger among students exposed to factory farm videos, extending previous research into the psychological basis of the effect. We also had two main exploratory questions. Do students exposed to the instructional material but not the videos also purchase less meat than the comparison group? And do TAs’ personal attitudes toward vegetarianism influence the results? If instruction can have an effect without the film and/or when the instructor is not vegetarian, that would show that the results of Schwitzgebel et al. (2020) did not depend on those aspects of the intervention. In contrast, if those features are necessary for an effect, that suggests limitations in the generalizability of that earlier research.

We had also hoped to examine longer-term data to look for effects that might have lasted a year or more. However, the COVID-19 pandemic interfered with data collection, preventing us from completing that part of the study.

## Method

### Participants and Instructors

Participants were 944 students enrolled in three introductory Philosophy courses at University of California, Riverside (UCR): 328 students in Philosophy 1 (Introduction to Philosophy), 300 students in Philosophy 2 (Contemporary Moral Issues), and 316 students in Philosophy 5 (Evil). Philosophy 5 was taught by one of the authors of this article (Schwitzgebel). The other classes were taught by other Philosophy Department faculty. Fifteen teaching assistants ran weekly discussion sections, with enrollment capped at 25 students per section (20 per section in Philosophy 5) and three sections per TA. Twelve of the TAs were Philosophy Department PhD students, two were pursuing a Master’s in Public Policy, and one was the principal instructor of Philosophy 5 for an honors section of 15 students.

### Design

During the first full week of class, September 30 to October 4, 2019, all students received an email link to a questionnaire containing twelve questions on four ethical issues, including the ethics of eating meat, which they could complete for a small amount of extra credit.

In their regularly assigned discussion sections during the week of November 4–8, 2019, students discussed a required reading arguing that it is unethical to eat factory farmed meat. Half of the section meetings began with a video advocating vegetarianism and containing graphic footage from factory farms (the film condition). TAs were encouraged to solicit and discuss students’ reactions to the video, using that as part of the basis of their philosophical discussion of the ethical issues. Half of the section meetings showed no video, and TAs were encouraged to keep the philosophical discussion relatively unemotional to the extent feasible (the non-film condition).

We did not otherwise attempt to control TAs’ teaching style, encouraging them to teach as much as possible in their usual style, with student learning as their overriding concern. We had two reasons for asking TAs to employ their ordinary instructional techniques. First, we wanted to see the effects of philosophical teaching as it occurs “in the wild”, so to speak, by normal TAs teaching in their usual way. Second, since students consented to be instructed but did not consent in advance to participating in an experiment, we did not want to compromise instructional aims by pressuring TAs to adopt teaching styles they might have judged to be less effective than their usual approach. We recorded whether the TA was a vegetarian and whether they revealed or did not reveal that fact to their students, but we did not attempt to record other aspects of teaching style.

Thirteen of the TAs taught either two film sections and one non-film section or one film section and two-non-film sections. One TA declined to show the film and so taught three non-film sections. One TA taught only one honors non-film section. Film vs non-film sections were approximately balanced by time of day and day of the week, and students were not told in advance the nature of the study or that a video was being shown in some but not all of the course discussion sections. In one Philosophy 2 section meeting, the film was shown but without sound, and the 25 students in that section were excluded from the study.

A few minutes before the end of each section meeting, students were given an opportunity to anonymously pledge to avoid eating meat for the next 24 h. TAs recorded the number of students attending and the number of students anonymously pledging in each section.

Within a few days after the last section meeting, students received an email link to a follow-up questionnaire containing the same twelve ethical questions as in the initial questionnaire, plus three additional questions.

We were able to obtain food purchase data for the minority of students (113 in all) who used their Student ID card for purchases at selected UCR on-campus restaurants from September 26, 2019, through January 13, 2020, allowing us to compare expressed opinion and actual food choice among that subset of students, before and after intervention, between the film and non-film conditions, and also against a comparison group of 226 students not enrolled in any of the three courses. For student privacy, all food purchase data and questionnaire responses were tagged to unique identifiers so that no individual student’s food purchase behavior or questionnaire answers could be known.

Our analyses rely on two-tailed statistical tests with an alpha level of .05. For the food purchase data and pledge data, the main hypotheses, overall method, and main statistical tests were pre-registered at AsPredicted. One exception is that we had hoped to have food purchase data from all of 2019. However, upon examination, the data before September, 2019, were too unsystematic to include in the study, due to irregularities as campus Dining Services transitioned between computer systems (see Supplementary Information Appendix A). Another exception is the doubling of the size of the comparison group, due to a lower than expected sample size for the treatment group (see below). All stimulus materials, pre-registrations, and data are available at https://osf.io/exg7f/?view_only=557d3f45ff554eaab9837cc7abf3b23b.

### Teaching Materials

Students were required to read James Rachels’ article “The Basic Argument for Vegetarianism” (Rachels 2004), an introductory-level ten-page philosophy article arguing that it is unethical to eat factory-farmed meat. In the film condition, at the beginning of section and before discussing any class content, TAs played the eleven-minute vegetarianism advocacy video “What Came Before” (http://whatcamebefore.com). Before showing the video, TAs informed the students that the video contained factory farm footage that some students might find upsetting, that it was optional, and that they would not be tested on its contents although its contents would serve as part of the basis for class discussion. Students who wished to opt out of viewing the video were invited to leave the room and return after eleven minutes. (In fact, only a single student left during the video presentation. We subsequently learned that some students chose to “opt out” by closing their eyes during a portion of the video, but this was not systematically recorded.) We chose “What Came Before” in part because earlier research suggested that it might be effective in encouraging viewers to explore vegetarianism or meat reduction (Cooney 2013; Schwitzgebel et al. 2020).

We did not assign “con” readings that argued that meat eating is permissible. TAs presented both pro and con considerations in their discussion sections, encouraging debate among students, as is typical procedure in philosophy discussion sections in the U.S. It is not unusual in philosophy instruction to assign material on only one side of a debate, especially if it expresses a philosophical position to which a majority of students are likely to be opposed, and then open up discussion of the pros and cons orally. The Rachels article is commonly used in ethics classes, and some classes (including the authors’ own non-experimental teaching) include optional video material on meat ethics. These materials are not unusual in the context of university-level philosophy instruction.

Students who did not attend their discussion sections that day were excluded from the study.

### Pledge Opportunity

Seven minutes before the end of each section meeting, TAs displayed a “pledge form” containing the following announcement, and a large blank space below, and they read the announcement aloud:

As part of an experiment by Professor Schwitzgebel (approved by UCR's research ethics review board), we are interested in knowing how many students in various sections are willing to pledge not to eat the meat of any factory farmed animals for the next 24 hours. The purpose of this research is to examine the effects of philosophical teaching on students’ opinions and food choices. In several days, you will have the opportunity to complete a questionnaire expressing your opinions about various moral issues, including the issue of eating meat.

Pledging is voluntary and will have no influence on your grade in this course.

At no point will your TA or Professor Schwitzgebel know whether you have pledged, though you will later have an opportunity to report whether you have pledged, in a way that will not reveal your identity to the professor or TAs.

By hand-drawing a figure in the space below, I am pledging not to eat the meat of any factory farmed animals for the next 24 hours. (Please do not write your name or any other identifying information.)

TAs also showed students a stack of 25 “pledge sheets” containing the statement “I PLEDGE NOT TO EAT THE MEAT OF ANY FACTORY FARMED ANIMALS FOR THE NEXT 24 HOURS” as well as contact information for University of California, Riverside’s Institutional Review Board.

The TAs then announced that they would be leaving the room while students decided whether to anonymously sign the pledge form with their doodle. Students were instructed to take a pledge sheet if and only if they drew a figure on the pledge form. The TAs then left the classroom, returning only after all students had left the room. They counted the number of doodles on the form and the number of missing pledge sheets. In cases where the number of doodles did not match the number of pledge sheets, the number of doodles was taken to be indicative of the number of students who had pledged. In one class, the pledge form was ignored so the pledge sheets were used as a count. In 27/41 of the remaining sections the doodle-based total and the sheets-based total matched exactly, and in no case did the totals differ by more than 3.

### Questionnaires

During the first week of instruction, Schwitzgebel emailed students in all courses, introducing himself as a UCR philosophy professor interested in students’ attitudes about ethical issues, contacting them with the permission of their instructor. (This language was modified for Philosophy 5, where Schwitzgebel was the instructor.) Students were told they could participate in a short questionnaire on four ethical issues for a small amount of extra credit in the course.

To help ensure confidence in anonymity and reduce demand (participants responding as they think the experimenter expects), the first page of the questionnaire contained the following language: “This study is being conducted by Professor Schwitzgebel, who is a philosophy professor here at UCR, with the permission of your instructor for Philosophy [XXX], Professor [XXX]. Neither your TA nor Professor [XXX] will be told your answers to these questions. All identifying information will be stripped from your answers before Professor Schwitzgebel views the answers, so that no one will know how any particular student has answered. You will not be graded on your particular answers, and you should feel free to disagree with your professor and TA about the ethical issues at hand.” (This language was simplified for Philosophy 5.) Recruitment emails contained similar assurances.

Several days later, a follow-up email reminded students about the extra-credit questionnaire. Instructors and TAs were also encouraged to remind students of the extra credit opportunity.

The main body of the initial questionnaire consisted of three questions on four topics, always in the same order: sexual intercourse outside of a committed, loving relationship; eating the meat of factory-farmed animals; spending a large amount of money on luxuries; and downloading music in violation of copyright laws. On each of the four topics, students were asked, again always in the same order, whether the behavior is unethical, whether they plan to avoid it, and whether if they engage in that behavior they should feel guilty. All responses were on a seven-point scale from “strongly agree” (+3) to “strongly disagree” (−3).

The three meat ethics questions were:

4. Eating the meat of factory farmed animals is unethical.
5. For at least the next month, I will eat no factory farmed meat at all – or if I find it too difficult to stick to that, I will eat it at most once per week.
6. If I eat factory farmed animals, I should feel guilty about that.

Each set of three questions appeared on a new page, with no opportunity for participants to view or correct responses from previous pages.

This questionnaire – the pre-test – was very similar to the questionnaire employed in Schwitzgebel et al. (2020), except that Question 5 was changed in hopes of getting a better correlation between responses to that question and measured meat purchases. (In Schwitzgebel et al. 2020, “I plan to choose non-factory farmed or vegetarian foods when they are available” correlated at only r = −.22 with the measure of meat purchases.)

A second questionnaire – the main questionnaire – was distributed one to five days after the discussion section meetings were completed in early November (thus five to six weeks after the initial questionnaire). The email prompts, reminders, and extra credit were similar to those in the initial questionnaire. The questions were identical except that three new questions were added to the end:

- 13. Did you watch the optional vegetarianism advocacy video “What Came Before?” that was shown at the beginning of some of the section meetings in which vegetarianism was discussed?
- [] I did not attend section that day.
- [] The video was not shown in my section.
- [] The video was shown in my section but I chose not to watch it.
- [] I started watching the video but didn’t finish.
- [] I watched the whole video.
- [] I prefer not to answer.
- 14. If you attended the section discussion meeting on meat ethics, did you take the pledge at the end of the section meeting?
- [] no
- [] yes
- [] did not attend section that day
- [] prefer not to answer
- 15. If you took the vegetarianism pledge at the end of the discussion section meeting, did you fulfill that pledge by not eating any factory farmed meat for the following 24 hours? (Any answer is okay, and neither the professor nor the TA will know how any individual student answered.)
- [] I did not take the pledge.
- [] I took the pledge but I ended up eating some factory farmed meat during the next 24 hours anyway.
- [] I took the pledge and I fulfilled the pledge by not eating any factory farmed meat during the next 24 hours.
- [] I prefer not to answer.

### Dining Card Data

A minority of University of California, Riverside’s students use their Student ID cards for on-campus purchases. UCR’s Dining Services team provided us with a complete list of all card purchases from February 1, 2019, through January 13, 2020 (the date of our data request). Unfortunately, data prior to Fall 2019 proved unusable, so analysis is confined to data starting September 26, 2019 (the first date of Fall Quarter). Matching Student ID data with purchase data also proved to be a somewhat complicated procedure, with room for a small amount of error, possibly up to 1% false positives and up to 5% false negatives. We explain the matching procedures in detail in Supplementary Information Appendix A. We then searched this database for the 730 students who had been enrolled in Philosophy 1, 2, or 5 in Fall 2019, excluding absent students and students in the section with the technical malfunction.

Based on rates of card use in Schwitzgebel et al. (2020), we had anticipated that about a third of students would have used their ID for purchases during the period, with an average of about 28 included purchases each. With 730 students, this would have given us 6813 purchases by 243 students, sufficient power for an 80% chance of detecting a decline from 28% to 25% meat purchases (odds ratio 0.86), even in a somewhat unbalanced design, comparable to the decline from 28.1% to 24.8% meat purchases (odds ratio 0.85) reported in Schwitzgebel et al. (2020). Unfortunately, however, only 13.6% of the included students, 113 total, used their ID cards for 2828 recorded purchases at the included campus restaurants, yielding a post-hoc power of 51% to detect an effect of the size reported in Schwitzgebel et al. (2020). However, with campus dining centers closed due to the pandemic, and given a scheduled transition to a new purchase-recording technology after eventual re-opening, we could not extend this protocol despite the lower-than-expected statistical power for the dining card data portion of the experiment. This mediocre power makes the confidence intervals reported below larger than ideal for detecting small effects of the sort expected and makes some of our null results difficult to interpret. However, it poses less of an interpretative problem for the positive results we report, especially given preregistration, which prevents running multiple underpowered studies or analyses and then illegitimately not reporting our null results to create the illusion of large effect. Power for the questionnaire and pledge portions of the experiment was not impaired by the low rate of card use.

We had originally planned to compare students in Philosophy 1, 2, 5 for whom we had purchase data with an equal-sized comparison group. However, due to the low number of students with available dining card data, we decided to double the size of the comparison group to reduce sampling error in the comparison group. Since Student ID numbers are approximately sequentially assigned based on date of first enrollment at University of California, Riverside, we chose comparison students with Student ID numbers adjacent to the Student ID numbers of the 113 target students, excluding students who were enrolled in Phil 1, 2, or 5 in Fall 2019, students who had participated in a pilot version of the study in Spring 2019, and students who were already serving as comparisons. (Those students were replaced with the next-nearest ID numbers.) This yielded a comparison group of 226 students for whom we had purchase data.

In accordance with our pre-registration, we planned to analyze the data both by examining all purchases and also by examining only purchases of $4.99 or more, to better target full-meal purchases.

All aspects of the design were pre-approved by University of California, Riverside’s Institutional Review Board (IRB-HS-18-170).

## Results

### Attendance and Exclusions

Of the 944 originally enrolled students, 25 were excluded from analysis due to a technical error in their discussion section (the video played but without sound), and another 189 were excluded for not having been present on the day of the discussion section, leaving 730 students for analysis, 275 (153 film) in Philosophy 1, 201 (75 film) in Philosophy 2, and 254 (130 film) in Philosophy 5, among which we had food purchase data for 113 (33, 38, and 42 in the three classes, respectively). As described above, the comparison group for the food purchase data consisted of an additional 226 students with numerically adjacent Student ID numbers.

### Questionnaires

The pre-test response rate was 81.2% (593/730), ranging from 73.1% in Philosophy 1 to 86.6% in Philosophy 5. The post-test questionnaire response rate was 78.5% (573/730), ranging from 73.1% in Philosophy 1 to 87.8% in Philosophy 5.

When correcting for multiple comparisons, students in the film condition and non-film condition did not detectably differ in their responses to any of the questions concerning the ethics of sex, luxury, and copyright, pre or post (|*t*| ≤ 2.1, *p* ≥ .04, 18 comparisons). Nor did students in the film condition detectably differ from students in the non-film condition on the pretest questions about the ethics of eating meat (|*t*| ≤ 1.6, *p* ≥ .10), with the largest difference on the question about avoiding eating meat for the next month (*M*_*film*_ = −1.09, *M*_*nonfilm*_ = −0.82, pooled *SD* = 1.94, *t*(591) = −1.64, *p* = .10, *d* = -.14).

**Fig. 1:**
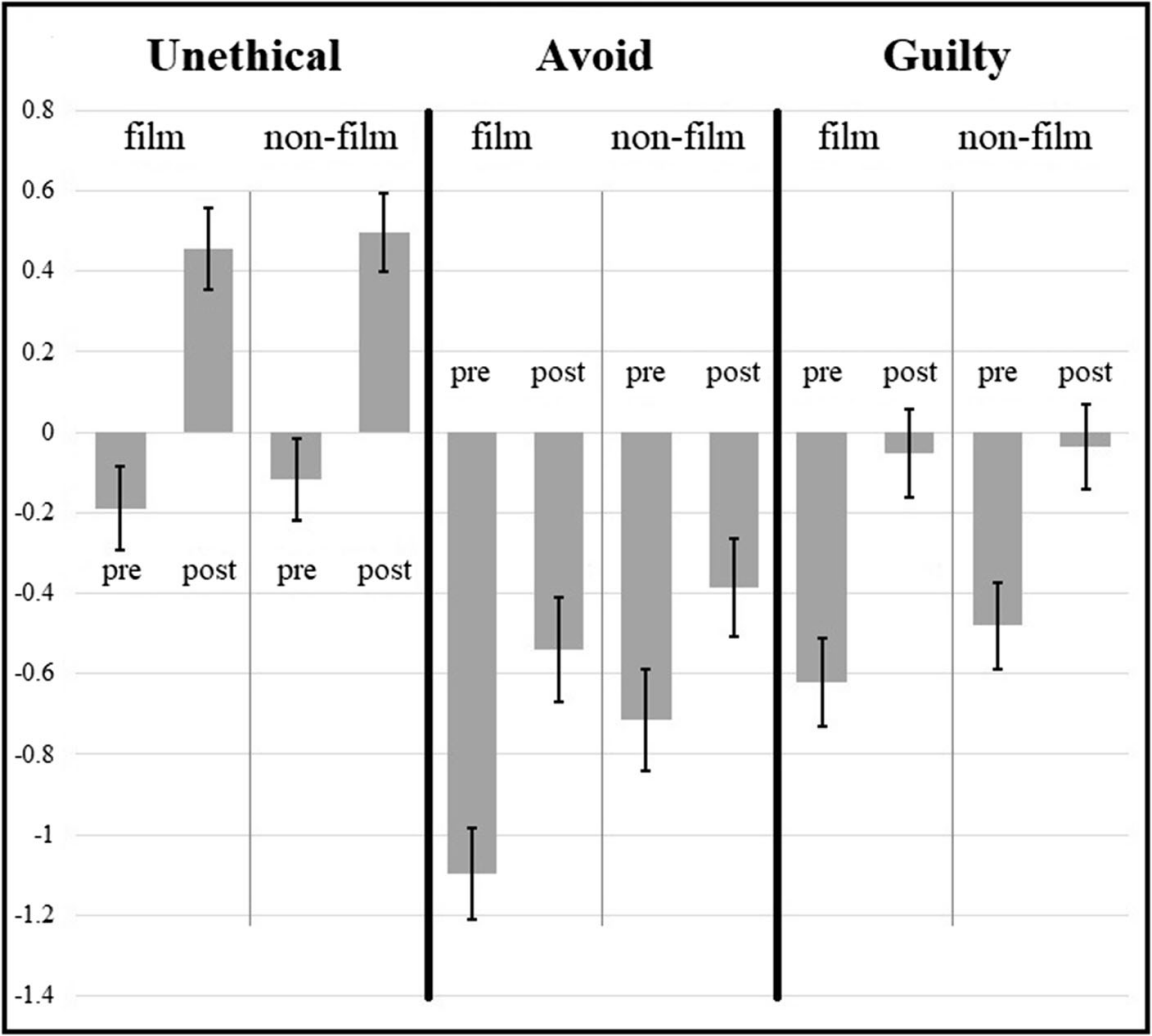
Mean agreement (+3 to −3) with three target questions (whether eating factory farmed meat is *unethical*, whether the respondent will *avoid* doing so, and whether the respondent should feel *guilty* if they do so), before (“pre”) vs. after (“post”) meat ethics instruction that either did or did not include a vegetarianism advocacy film with factory farm footage, among students who responded to both questionnaires. Error bars are ± 1 standard error

On all three of the meat ethics questions, students expressed significantly more agreement after the discussion meetings than they did at the beginning of the term. The shifts of opinion were substantial and not much different in the film condition than in the non-film condition. For example, in the film condition, in the pre-test 35% of students agreed that eating the meat of factory farmed animals is unethical, compared to 51% after (98/279 vs. 143/278, two-proportion *z* = 3.94, *p* < .001, *φ* = .16). In the non-film condition, 37% agreed before, compared to 56% after (116/314 vs. 165/295, two-proportion *z* = 4.78, *p* < .001, *φ* = .19). On the main opinion question, “Eating the meat of factory farmed animals is unethical”, tests for differences between the film and non-film condition, post-intervention, showed no statistically detectable effects between the conditions (143/278 vs. 165/295, two proportion *z* = −1.08, p = .28, preregistered; *M*_*film*_ = +0.46, *M*_*nonfilm*_ = +0.49 pooled *SD* = 1.62, *t*(571) = −0.20, p = .84, preregistered; post-hoc power analysis: 80% chance of detecting an effect size of *d* = .23). Students also responded similarly to “avoid” and “guilty” questions regardless of exposure to the film (avoid: *M*_*film*_ = −0.59, *M*_*nonfilm*_ = −0.38, pooled *SD* = 2.03, *t*(571) = −1.22, p = .22; guilty: *M*_*film*_ = −0.07, *M*_*nonfilm*_ = −0.01, pooled *SD* = 1.77, *t*(571) = −0.37, p = .71). See also Table 1 and Fig. 1. Supplementary Information Appendix B summarizes results of similar questionnaires in four classes from Fall 2018 through Spring 2019.

**Table 1:** Mean agreement (+3 to −3 agree/disagree scale) and percentage agreement (“slightly agree” (+1) or higher) with three claims about meat ethics, among students who responded to both questionnaires (N = 518)

Question	Pretest			After Intervention			*d*	Test Statistic	
	*M*	*SD*	%agr	*M*	*SD*	%agr		paired *t*	*p*
“Eating the meat of factory farmed animals is unethical.”	−0.15	1.66	37%	+0.48	1.61	54%	.43	9.67	< .001
“For at least the next month, I will eat no factory farmed meat at all – or if I find it too difficult to stick to that, I will eat it at most once per week.”	−0.90	1.95	27%	−0.46	2.01	34%	.27	6.08	< .001
“If I eat factory farmed animals, I should feel guilty about that.”	−0.55	1.75	32%	−0.04	1.74	42%	.32	7.28	< .001

To test for differences by class and TA, we created a composite meat ethics score by averaging the three meat ethics responses in the post-test. The three classes did not detectably differ in their composite score, ranging from a mean of −0.15 for Philosophy 1 to +0.11 for Philosophy 5 (ANOVA, *F*(2, 570) = 1.57, *p* = 0.21). Nor did we detect differences by TA, with composite scores ranging from −0.42 to +0.53 (ANOVA, *F*(13, 546) = 1.34, *p* = 0.18 [excluding the honors section TA due to small sample size, *M* = +0.49]). Respondents whose TAs were vegetarian (N = 177) did not detectably express more agreement with the meat ethics questions than did respondents whose TAs were not vegetarian (N = 357) (*M*_*TAveg*_ = +0.11, *M*_*TAnonveg*_ = −0.10, pooled *SD* = 1.55, *t* = 1.48, *p* = .14; excluding respondents from one TA who reported being nearly vegetarian). Responses did differ somewhat if the TA explicitly *revealed* that they were vegetarian (N = 81) or non-vegetarian (N = 124) (*M*_*revealveg*_ = +0.32, *M*_*revealnonveg*_ = −0.19, pooled *SD* = 1.58, *t* = 2.25, *p* = .025, *d* = .32). However, even among students whose TAs revealed that they were non-vegetarian, students’ composite attitudes were significantly different pre vs. post, both in the film and in the non-film conditions (film: *M*_*pre*_ = −0.67, *M*_*post*_ = −0.30, *SD* of diff = 1.04, paired-*t*(86) = 3.28, *p* = .002, *d* = .35; non-film *M*_*pre*_ = −0.27, *M*_*post*_ = +0.26, *SD* of diff = 1.27, paired-*t*(59) = 3.25, *p* = .002, *d* = .42).

### Pledge Opportunity

Overall, 751 students were recorded as having attended section on the day of discussion. This is somewhat higher than the 730 students included in the other portions of the study because it includes students who added the course after the first week, for whom we didn’t have enrollment information. In the film condition, 36% of students pledged, compared to 41% pledging in the non-film condition, a statistically non-significant difference, against the direction of our hypothesis (141/388 vs. 150/363, two-proportion *z* = −1.40, *p* = .16, preregistered).

Looking at self-report of pledging among questionnaire respondents, overall, 43% (248/573) of respondents with recorded section attendance reported having pledged to avoid eating meat for 24 h and 33% (189/573) reported having kept that pledge. These results did not detectably differ between the film and non-film conditions (pledged: 110/278 film vs. 138/295 non-film, two-proportion *z* = −1.75, p = .08, preregistered; kept pledge: 86/278 film, 103/295 non-film, two-proportion *z* = −1.01, p = .31, preregistered). Unsurprisingly, those who reported pledging expressed more agreement with the claim that eating the meat of factory farmed animals is unethical than did those who reported not having pledged (*M*_*pledged*_ = 1.19, *M*_*notpledged*_ = −0.12, pooled *SD* = 1.48, *t*(546) = 10.32, *p* < .001, *d* = .89; 75% (185/248) vs. 37% (111/300), two-proportion z = 9.58, p < .001, *φ* = .38).

There was considerable heterogeneity in pledge rates between sections, with pledge rates ranging from 5% (1/19) to 79% (11/14; *SD* of percentages = 0.19; *χ*^*2*^[41] = 112.7, *p* < .001, *φ* = .39; since one cell had an expected value of 4.3, we confirmed with a Monte Carlo redistribution, again finding p < .001). TAs who were vegetarian had similar pledge rates to those who were non-vegetarian (40% vs. 37%, 93/235 vs. 174/472, *z* = 0.70, *p* = .49; excluding the nearly vegetarian TA, whose pledge rate was 55%, 24/44). However, we interpret this non-effect cautiously given the modest sample size (95% CI = −5% to +10%) and the presence of a small effect in the predicted direction in the larger sample discussed in Supplementary Information Appendix B, which contains pledge data from similar designs in four classes from Fall 2018 through Spring 2019. Similar to the results on attitude, pledging appears to have been higher for students whose TAs explicitly revealed that they were vegetarian than those who revealed they were non-vegetarian (31% vs. 20%, 37/120 vs. 28/142, *z* = 2.07, *p* = .04, *φ* = .13), a result confirmed in Supplementary Information Appendix B.

Post-hoc, some TAs reported apparent group effects. For example, one TA reported that before she was able to leave the room, one student stood up and announced that they would pledge, which seemed to encourage other students also to commit to pledging. We conclude that the in-classroom behavior of choosing to pledge or not pledge while peers observe is likely influenced by features of the group and/or situation that we were unable to control or model in this experiment.

### Purchase Data

#### By Purchase

We matched 2828 purchases to 113 students (56 film, 57 non-film) in Philosophy 1, 2, and 5 who used their ID card for food purchases on campus. We matched 5033 purchases to 226 comparison students with adjacent Student ID numbers. Of these purchases, 48% (3742/7861) were purchases of at least $4.99, and 41% (3227/7861) were on or after the day of the discussion section (or November 6 for the comparison group of 226 students who were not enrolled in any of the three courses).

Table 2 shows the percentage of meat purchases for the treatment groups and the comparison group, before and after the day of the section meeting (or Wednesday, November 6, for the comparison group). As is evident from the table, before the day of the discussion section meeting, meat purchase rates were similar among the comparison group and the film and non-film conditions in the treatment group. After the day of the discussion section meeting, meat purchases rates remained virtually identical in the comparison group and declined sharply among students in the film condition, from 30% to 21% of purchases overall and from 51% to 37% of purchases over $4.99. Students in the non-film condition showed intermediate effect sizes, including a barely statistically significant decline in their overall rate of meat purchases.

**Table 2:** Percent meat purchases as measured from dining card receipts, before and after discussion of meat ethics (treatment group) versus no intervention (comparison group), purchase-by-purchase analysis (all tests preregistered)

	% Meat Purchases		Test Statistics			
Condition	before	after	*z*	*p*	95% CI for diff	odds ratio
Comparison group						
all purchases (N = 5033)	30.7%	30.4%	0.27	.79	−3.0% to +2.3%	0.98
$4.99 or more (N = 2399)	52.0%	53.0%	−0.49	.62	−3.1% to +5.2%	1.04
Treatment group, all purchases						
film condition (N = 1418)	29.6%	20.9%	3.82	<.001	−13.3% to −4.3%	0.63
non-film (N = 1410)	29.8%	25.1%	1.98	.047	−9.4% to −0.1%	0.79
*total*	*29.7%*	*23.0%*	*4.05*	*<.001*	*−9.9% to − 3.5%*	*0.71*
Treatment group, $4.99 or more						
film condition (N = 671)	51.4%	36.7%	3.83	<.001	−22.2% to −7.2%	0.55
non-film (N = 672)	50.0%	46.8%	0.82	.41	−10.8% to +4.5%	0.88
*total*	*50.7%*	*41.8%*	*3.25*	*.001*	*−14.3% to − 3.5%*	*0.70*

Meat purchases were typically full meals (e.g., chicken and bacon sandwich with chips). Non-meat purchases were a mix of snack items, expensive drinks, and full vegetarian meals (with about 2% non-food purchases), not always fully distinguishable. Although we cannot share the full proprietary data on purchase items, a random sample of seven non-meat items should give the reader a sense of the purchase types: cheese quesadilla; non-caffeinated large drink; water and candy items; large frappucino; large veggie snack box and chai tea; large macchiato; vegan garden burger. Post-hoc inspection of the data suggested that vegetarian entrees may have been more likely than meat entrees to cost less than $4.99 (e.g., grilled cheese sandwich, hummus sandwich, PB&J sandwich, all $2.99–$3.49). If meat is also sometimes replaced by increased snacking, this would also problematize the $4.99 cutoff.

#### By participant

We also examined purchase data participant-by-participant. This allowed us to assess whether the purchase-by-purchase results might have been driven by just a few participants. Of the 339 students with at least one included purchase, 294 had recorded purchases both before and after the intervention (49 in the film condition, 50 in the non-film condition, and 195 in the comparison group) and 261 had recorded purchases of at least $4.99 both before and after the intervention (40 in the film condition, 47 in the non-film condition, and 174 in the comparison group). The unexpectedly small number of participants in the film and non-film conditions creates potential problems with statistical power in participant-level analyses but at the same time makes it important to run participant-level analyses in case chance differences among participants explain the purchase-level effects (analysis of which assumes that the purchases are statistically independent).

We employed a pre-registered multilevel logistic regression including all 339 participants, predicting whether a purchase contained meat (0 = vegetarian, 1 = meat) from a variable that was 1 if the purchase was made after instruction by a student who received the meat ethics instruction and 0 otherwise, both for all purchases and for purchases of $4.99 or more. To examine results by condition, we reran the first analysis twice, each time excluding students from the opposing condition. The results are displayed in Table 3.

**Table 3:** The odds of a meat purchase in the treatment group after intervention, compared to the odds of a meat purchase in the comparison group or in the treatment group before intervention, with participant as a random effect (all tests preregistered)

Condition	odds ratio	95% CI	*p*
All treated participants			
all purchases (N = 7861)	0.73	0.61–0.87	< .001
$4.99 or more (N = 3742)	0.69	0.54–0.89	.005
Film participants only vs. comparison group			
all purchases (N = 6451)	0.68	0.52–0.88	.003
Non-film participants only vs. comparison group			
all purchases (N = 6443)	0.77	0.60–0.99	.042

Thus, an analysis that accounts for possible participant-level failures of independence confirms the results of the simpler purchase-by-purchase analysis. Nevertheless, given the modest *p* value for the decline among the non-film participants, caution is warranted. To check robustness, we also ran the analyses in Table 2 limiting to just the 294 participants with recorded purchases both before and after intervention. The results were essentially the same, including a similar near-threshold *p* value of .035 for the decline in total meat purchases for the non-film participants.

We were also curious if we could find any evidence of vegetarianism among our participants. Among the students in the treatment conditions, 83 had at least five recorded purchases of $4.99 of more, and among those, 20 had less than 20% meat purchases after intervention. We examined every transaction by these 20 students. Three of these students (all in the non-film condition) had several purchases that were clearly full vegetarian meals both before and after the intervention. They were likely practicing vegetarians or nearly so, giving us a rough estimate of 4% vegetarians among UCR students enrolled in lower-division philosophy classes. (This fits with post-hoc reports by some TAs that about 0 to 2 students per section meeting said they were vegetarian.) Three other students (two film, one non-film) had meat purchases before intervention and at least one full vegetarian meal after intervention. However, none of these students had enough recorded post-intervention full meal purchases for us to confidently infer that they converted to vegetarianism for any extended period. The remaining 14 students with under 20% meat purchases had mostly expensive drink and snack purchases, either with some meat meals mixed in or without full meals of the sort that would enable us to infer vegetarianism.

#### By TA Attitude

TA attitude had no detectable effect on the purchase results. Among students whose TAs were vegetarian, meat purchases declined from 30% before intervention to 23% after intervention (154/516 vs. 106/468, *z* = 2.58, *p* = .010, *φ* = .08; excluding purchases under $4.99, 46% vs. 38%, 136/297 vs. 83/220, *z* = 1.85, *p* = .065, *φ* = .08; again, excluding respondents from one TA who reported being nearly vegetarian). Among students whose TAs were not vegetarian, meat purchases declined from 28% before intervention to 23% after intervention (277/994 vs. 182/797, *z* = 2.45, *p* = .014, *φ* = .06; excluding purchases under $4.99, 51% vs. 43%, 231/454 vs. 145/334, *z* = 2.08, *p* = .037, *φ* = .07). Not only was there a significant decrease in meat purchases among students with non-vegetarian TAs, but also effect sizes were similar for participants with vegetarian and non-vegetarian TAs. Looking only at students with non-vegetarian TAs who explicitly revealed their non-vegetarian behavior, we see a similar but statistically non-significant decline from 27% before intervention to 22% after (*z* = 1.60, *p* = .11, 95% CI for diff = −12% to +1%; film: 65/242 before vs. 43/198 after; non-film: 41/144 before vs. 28/121 after). As is evident from the large confidence interval, the sample size is too small to draw a confident conclusion about the presence or absence of an effect of the expected magnitude in this particular subgroup.

#### Relationship with Expressed Attitudes

As Schwitzgebel and collaborators also found in earlier research (Schwitzgebel and Rust 2014; Schwitzgebel et al. 2020), relationships were generally modest between expressed ethical attitudes about meat eating and measured behavior. Among participants who agreed that eating meat is unethical, 20% of recorded purchases after intervention contained meat, compared to 26% of purchases among those who did not agree that eating meat is unethical (98/487 vs. 151/587, *z* = 2.19, *p* = .029, *φ* = .07; excluding purchases under $4.99, 35% vs. 50%, 81/229 vs. 120/238, *z* = 3.32, *p* = .001, *φ* = .15). Even among participants who agreed that they would avoid eating meat for at least the next month, 17% of their purchases after intervention contained meat, compared to 26% of purchases among those who did not agree that they would avoid eating meat (53/308 vs. 196/766, *z* = 3.14, *p* = .002, *φ* = .09; excluding purchases under $4.99, 32% vs. 47%, 41/127 vs. 160/340, *z* = 2.98, *p* = .003, *φ* = .13). These small effect sizes highlight the value (also emphasized in Peacocke 2018) of employing direct measures of behavior when studying vegetarian vs. non-vegetarian food choice, as opposed to self-reported attitude or intention.

## Conclusion

Replicating Schwitzgebel et al. (2020), students exposed to standard philosophical instructional materials on the ethics of eating meat substantially reduced their rate of meat purchases at campus dining locations, from 30% of purchases overall to 23%, and from 51% to 42% if only purchases of at least $4.99 are considered. In view of similar results also from Jalil et al. (2020), it seems clear that ordinary classroom instruction on the ethics of eating meat can influence students’ practical day-to-day choices. Students purchase less meat after being exposed to material on meat ethics. In this one respect at least, philosophical instruction in the classroom can substantially influence students’ real-world behavior. All three studies were conducted among southern California university students, so it remains unclear how far the results would generalize to other cultural contexts. Nonetheless, given the general absence of good evidence of *any* effects of university-level philosophy instruction on real-world behavior outside the classroom, even showing a local effect is a valuable existence proof.

The magnitude of the effect is notable. Although the decline in meat purchases from 30% to 23% (or 51% to 42%) is in a certain sense small, in another sense it constitutes a substantial change in behavior after a relatively brief intervention. It would have been reasonable to suspect that university students’ meal purchase patterns would be stable over time and resistant to change on the basis of classroom discussion. Contrary to that suspicion, it appears that material change is possible, at least over the time period of available data – several weeks to several months, in the three studies discussed. Although few if any students were converted immediately to strict vegetarianism, many were ready to somewhat decrease their meat consumption.

Also replicating Schwitzgebel et al. (2020), students’ expressed attitudes about meat ethics also shifted substantially in response to instruction. In an anonymous questionnaire during the first week of instruction, before discussion of any meat ethics material, 37% of students agreed that eating the meat of factory farmed animals is unethical, compared to 54% in an anonymous questionnaire after instruction. This is in our view quite a substantial shift in expressed opinion, given that this is a topic that is generally already familiar to most university students in southern California. The anonymous questionnaires were designed to minimize experimenter demand and socially desirable responding (participants giving responses they think would be socially approved of). However, such factors probably almost inevitably play a role in self-report of moral attitudes and behavior. This is perhaps especially so for moral behavior, which seems to be especially driven by social conformity (Cialdini et al. 2006; Bicchieri 2017; Schwitzgebel 2019a).

In several important respects, this experiment extends the results of Schwitzgebel et al. (2020). First, while Schwitzgebel et al. (2020) used only vegetarian TAs, in this study the majority of TAs were non-vegetarian. Meat purchases declined significantly among participants with non-vegetarian TAs, and they did so with approximately the same estimated effect size as among participants with vegetarian TAs. The research thus suggests that Schwitzgebel, Cokelet, & Singer’s use of only vegetarian TAs was not necessary for producing their observed effect.

Second, we added an entirely new intervention and dependent measure, the pledge opportunity. We were struck by the effectiveness of the pledge opportunity. When given the opportunity to anonymously pledge to avoid eating factory farmed meat for 24 h, almost half of students took the pledge, and the majority of those reported having kept the pledge. If the self-reports are accurate, the pledge opportunity convinced 33% of students to refrain from eating factory farmed meat for 24 h, in a student population that is probably under 5% vegetarian. Instructors interested in persuading students to briefly try a vegetarian diet might wish to adopt this method. If students can be effectively encouraged to try vegetarianism short term, this might facilitate their later considering a long-term change in their dietary habits. A pledge opportunity of this sort is of course a type of social demand on students in the context of classroom instruction on meat ethics. The anonymity of the pledge presumably somewhat reduced demand or social pressure, and we found no evidence in the main study of a relationship between TA attitude and pledge rate, though extended data in Supplementary Information Appendix B suggested that students of TAs who were vegetarian were slightly more likely to pledge, especially if the TAs revealed their own vegetarian or non-vegetarian behavior. Social pressure from peers may have played a role in pledging, as also suggested by the high variability in pledge rates by section and the peer conformity informally observed by some of the TAs.

Third, Schwitzgebel et al. (2020) gave all participants the opportunity to watch a vegetarianism advocacy video, which the majority of participants reported at least starting to watch. This raises the prospect that behavioral change was caused mostly or wholly by exposure to the video rather than other aspects of instruction. In contrast, we presented the video to only half of the students. We hypothesized that instruction would be more effective when accompanied by the video, with vivid factory farm footage that many people find emotionally engaging. We also thought it possible that instruction would be largely ineffective without the video. Results on this question were mixed.

Expressed opinion and pledge rates did not differ between the film and non-film conditions. In fact, the pledge rates were non-significantly in the opposite direction of our hypothesis. Somewhat to our surprise, the condition involving reading and fifty minutes of relatively unemotional discussion was at least as effective in shifting expressed opinion and pledge rates as was the condition involving reading plus an eleven-minute video and thirty-nine minutes of discussion. We regard this as encouraging. Evidently, philosophy instructors need not lean on engaging videos to influence students. Reading and discussion might be just as effective.

When purchase behavior is directly measured, the interpretation is less clear. Students in the film condition showed a substantial and highly statistically significant change in their purchase behavior, declining from 30% to 21% meat purchases overall, with an odds ratio of 0.73 in an analysis that accounts for participant-level failures of statistical independence by treating participant as a random effect (both p’s < .001). In the non-film condition the estimated effect sizes were somewhat smaller and statistically less secure: a decline from 30% to 25% (p = .047) and an odds ratio of 0.77 (p = .042). When purchases are limited to those of $4.99 or more the statistical power collapses and no effect is statistically detectable in the non-film condition. Likewise, sample sizes were too small to permit meaningful statistical examination for interaction effects between TA attitude and film condition. Regrettably, as explained in the methods section, statistical power was limited due to lower-than-expected card use and the unusability of a portion of the database. Nonetheless, we are inclined to interpret the results as showing an effect in the non-film condition on the following grounds: First, the effect directions and statistical tests were pre-registered, and they did in fact cross the pre-registered alpha threshold of .05. And second, it would be slightly odd (though only slightly) to find the non-film condition to be as effective as or more effective than the film condition on the pledge and questionnaire measures but completely ineffective in the direct behavioral measure. In fact, the estimated effect size on purchases in our non-film condition, OR = 0.77, was larger than the OR = 0.85 reported in Schwitzgebel et al. (2020). The evidence thus appears to show that exposure to the film is not necessary for substantial behavioral change.

This study extends and conceptually replicates Jalil et al. (2020) and Schwitzgebel et al. (2020), showing that it is possible to influence students’ attitudes and daily behavior through standard methods of university-level philosophy instruction. As large and systematic databases of behavior become more accessible to researchers – within the constraints of student privacy – it will become increasingly possible to examine outside-the-classroom effects of instruction rigorously, without relying on inferences from behavior in artificial laboratory environments or on possibly misleading self-reports.

## Supplementary Information

ESM 1 (DOCX 19.5 kb)

ESM 2 (DOCX 20.7 kb)

ESM 3 (DOCX 12.5 kb)

ESM 4 (DOCX 12.1 kb)

## Data Availability

Included as Appendices G, H, and I at https://osf.io/exg7f/?view_only=557d3f45ff554eaab9837cc7abf3b23b.

## Appendix A: Further Methodological Details and Rank Analysis

### Purchase Data Methodological Details

A minority of University of California, Riverside’s students use their Student ID cards for on-campus purchases. On January 13, 2020, UCR’s Dining Services provided us with every Student ID card purchase from February 1, 2019, through that date. However, on examination, the data before September 26, 2019 (the first day of Fall Quarter instruction), were excluded as too unsystematic for straightforward analysis, due to large patches of non-randomly missing or misdated data during the transition in Dining Service computer systems in spring and summer 2020. For example, a campus Starbucks location, with mostly vegetarian purchases, provided much more complete data in spring than winter. This unsystematicity also prevented us from completing our plan to include data from students in two courses in Spring 2019. (Preliminary analysis of purchase data from students from Spring 2019 indeed showed fewer meat purchases after intervention than before intervention, in accord with our hypothesis, but this might have been due to the Starbucks effect or other unsystematicities in the data.) Appendix B presents questionnaire and pledge data for Spring 2019 students, as well as students from two other earlier courses in Fall 2018 and Winter 2019.

**Table 4:** Mean agreement (+3 to -3 agree/disagree scale) and percentage agreement (“slightly agree” (+1) or higher) with “Eating the meat of factory farmed animals is unethical” in four large lower division courses (all tests preregistered)

	non-film condition			film condition				
Course	*M*	*SD*	%agr	*M*	*SD*	%agr	*t*	*p*
Phil 5 F18	+0.15	1.76	47%	+0.68	1.66	60%	2.60	.010
Phil 2 W19	+0.47	1.72	59%	+0.35	1.59	51%	0.53	.60
Phil 1 S19	+0.36	1.74	57%	+0.79	1.33	68%	1.98	.049
Phil 2 S19	+0.48	1.43	50%	+0.20	1.62	43%	1.30	.20

Analysis required matching two different data sources. This was done before we discovered the unsystematicity in the data from early 2019. One data source was the food service software provider Appetize, which provided, for some but not all campus restaurant locations, date and time of purchase (to the minute), purchase location, purchase total, payment method, and the list of items purchased. This data source did not include Student ID. The second data source was student data software provider Blackboard, which provided Student ID, date and time of purchase (to the minute), purchase location, and payment method if the student paid with “Dining Dollars” or “Bear Bucks” on their Student ID card, but it did not include a list of items purchased. Appetize Data were reduced to include only purchases with Dining Dollars or Bear Bucks. Dining locations that did not itemize purchase details were excluded from both data sets. We looked for purchases that exactly matched purchase amount and time to the minute in both data sets. By this method, 85% of transactions matched. The match was confirmed by checking that the dining location and payment method also matched and by checking for double matches. Mismatches or double matches constituted 1.3% of the data, and were subsequently excluded. Examination of non-matched data suggested unsystematic clock asynchronies, so unmatched data were rematched based on purchase price and time rounded to 1/100 of a day (about 14 minutes). By this procedure, 66% of the previously unmatched transactions were matched. Examination of unmatched data suggested that most of the unmatched data were from the first half of 2019, which was our first evidence of unsystematicity. In the second round, mismatched vendor or payment method were 11% of the data (there were no double matches), which were then excluded. After all exclusions, for the Fall 2019 data, we estimate a false negative rate of at most 5% and a false positive rate of at most 1%. Student ID was then replaced with a unique identifier to preserve student privacy, creating a dataset containing each student’s unique identifier code, transaction date and time, purchase price, and items purchased.

In accord with our pre-registration, each transaction was then coded as red meat (3), poultry (2), seafood (1), or vegetarian (0), with the entire transaction coded based on the highest-value menu item. We planned both parametric analysis based on rank and categorical analysis of meat (1-3) versus vegetarian (0). However, as in Schwitzgebel, Cokelet, and Singer (2020), the parametric rank analysis added no important information to the simpler categorical analysis. For simplicity, we report the parametric rank analysis below in this appendix rather than in the body of the article.

Given the number of transactions, it was not possible to hand-code every transaction, so transactions were coded based on search terms such as “chicken”, “hamburger”, and “shrimp”. The entire list of purchases was alphabetized and skimmed to see what items were commonly listed and what abbreviations were commonly used (e.g., “ckn” for chicken), and to eliminate purchases containing only non-food items (e.g., blue books or medicines) and purchases whose vegetarian status could not be determined (e.g., “chefs special”). The list was then randomized and a sample was checked for coding errors (e.g., “veggie burger” erroneously classified as red meat due to containing “burger”), indeterminable purchases, and non-food purchases, leading to recoding and exclusions. This process was iterated until sampling suggested about 2% non-food or indeterminate items and < 1% coding error. All of this was completed before any analysis and without knowledge of which purchases were completed by students in the target classes.

### Rank Analysis

Rank analysis of score from 0 (vegetarian) to 1 (seafood) to 2 (poultry) to 3 (red meat) yielded similar results to the results reported in the main body of the article. The comparison group had a mean score across all purchases of 0.74 before intervention vs. 0.73 after (pooled *SD* = 1.17, *t*(5031) = 0.36, *p* = .72, preregistered), and for purchases of $4.99 or more the mean scores were 1.25 before and 1.29 after (pooled *SD* = 1.30, *t*(2397) = -0.64, *p* = .53, preregistered). In the film condition, mean scores declined from 0.76 to 0.55 overall and from 1.31 to 0.97 among purchases of $4.99 or more (pooled *SD* = 1.17, *t*(1416) = 3.36, *p* = .001, *d* = .18, preregistered; pooled *SD* = 1.34, *t*(669) = 3.23, *p* = .001, *d* = .25, preregistered). In the non-film condition, mean scores overall showed a statistically marginal decline from 0.70 to 0.59 (pooled *SD* = 1.10, *t*(1408) = 1.91, *p* = .056, *d* = .10, preregistered) and showed no appreciable decline among purchases of $4.99 or more (*M*_*before*_ = 1.17, *M*_*after*_ = 1.10, pooled *SD* = 1.25, *t*(670) = 0.72, *p* = .47, *d* = .06, preregistered). Given that the rank analysis did not add new useful information and is also less readily interpretable than the categorical analyses, we have de-emphasized it in our reporting and interpretation.

### Consent and Privacy

This study raises ethical concerns regarding consent and privacy that we wish to acknowledge here. Students did not consent to be sorted into sections that either viewed or did not view the film. However, films of this sort are sometimes used in philosophy instruction, and it is not unusual in philosophy courses to have one’s ethics challenged and to read and view potentially upsetting materials. Students in the film condition were told in advance that the film might be upsetting, that it was optional, and that they would not be tested on the material. Having different sections taught with different pedagogical techniques is within normal philosophical teaching practice, and we emphasized to the TAs that their duty was just their ordinary pedagogical duty: They were to teach the assigned topics as they would teach any other assigned topics, in accord with their usual teaching standards and techniques, with student learning as their overriding goal.

Students were not informed that their Dining Card purchases were subsequently examined. Students were not debriefed on this aspect of the study because the benefits of debriefing were judged not to outweigh the costs and risks. The costs would have included inability to conduct follow-up studies and the risks included the possibility of students falsely inferring violations of privacy. In all portions of the study, student privacy was protected by replacing student names with unique identifiers so that no individual’s purchases or questionnaire responses could be known. We also note that meal purchases in restaurants don’t normally come with a high expectation of privacy, since the food purchase is known to the cashier, visible to passersby, and recorded by the company that manages the card transactions. It is not unusual for companies to study consumer choice under various conditions without explicit consent.

## Appendix B: Participants from Earlier Courses

We also ran versions of this study with a similar design in one course in Fall 2018 (Philosophy 5 “Evil”), one in Winter 2019 (Philosophy 2 “Contemporary Moral Issues”), and two in Spring 2019 (Philosophy 1 “Introduction to Philosophy” and Philosophy 2 again, with a different syllabus and instructor). We had hoped to obtain good purchase data from students in those quarters, but that proved impossible. (A vendor change from Oracle to Appetize in early 2019 made the Fall 2018 data unobtainable and the Winter and Spring 2019 data irregular, with operations not fully smoothed out until Fall 2019.)

The questionnaire and pledge results from these four earlier courses were similar to those reported in the main study, with the exception that there was some evidence for course-level effects. We present summary results here for completeness and to show the replicability of our questionnaire and pledge results, and also because the possible course-level effects are potentially of interest.

### Questionnaire Results

The pre/post questionnaire format was introduced in the Spring 2019 version of the study. Of the 367 students who attended on the day of the discussion and completed both the pre and post questionnaires 33% (122/367) agreed (+1 to +3) that “eating the meat of factory farmed animals is unethical” on the pre-test, compared to 55% (202/367) after instruction, with a shift in mean response from -0.31 to +0.45 (paired *t* = 9.49, *p* < .001). This shift is similar to the shift from 37% to 54% agreement (mean -0.15 to +0.48) that we found in the main study, confirming that effect and showing that it was not confined to the three particular courses included in the main study.

To look for differences by film vs. non-film condition, we included all four courses. Among 916 discussion section attendees who responded to the post-instruction questionnaire, 55% (500/916) agreed that eating factory farmed meat was unethical – a result that did not detectably differ between the film and non-film conditions: film 57% (258/456), non-film 53% (242/460) (*z* = 1.21, *p* = .23; *M*_*film*_ = .53, *M*_*nonfilm*_ = .35, *t*(914) = 1.62, pooled *SD* = 1.63, *p* = .11). Results by course are displayed in Table 4 below. As is evident from the table, in two courses students might have been more likely in the film than in the non-film conditions to agree that eating factory farmed meat is unethical. However given the multiple comparisons, modest *p* values, and opposite-direction estimates in the other two courses, this should be interpreted cautiously.

To further test for heterogeneity among the courses, we ran a multiple regression analysis predicting agreement on the agree/disagree scale from dummy variables for condition (0 = non-film, 1 = film), course (each course a separate 0,1 dummy, with Phil 5 as the reference group), and the three interaction variables for course * film. This analysis finds film to be positively predictive (*β* = .16, *p* = .006), no statistically detectable effect by course, and statistically detectable negative interaction effects for two of the three courses Phil 2 Spring * film (*β* = -.15, *p* = .007) and Phil 2 Winter * film (*β* = -.13, *p* = .027). The interaction effects suggest that the exposure to the film had different results in the four classes. As suggested both by this analysis and by Table 4 above, exposure to the film may have increased agreement with “Eating the meat of factory farmed animals is unethical” in Philosophy 5 and Philosophy 1 (Evil and Introduction to Philosophy) but not in either of the two versions of Philosophy 2 (Contemporary Moral Issues). However, we regard this analysis as exploratory and uncertain.

### Pledge Results

Across the four courses, 565 students attended the film condition discussion sections (including three who opted out of viewing by exiting the room) and 573 attended the non-film discussion sections. Overall, the pledge rates are comparable to those in the main study (which found 36% pledging in the film condition and 41% in the non-film condition). In the four classes studied from Fall 2018 through Spring 2019, 237 (42%) pledged in the film condition, compared to 200 (35%) in the non-film condition, a statistically significant difference in the predicted direction (*z* = 2.45, *p* = .014, preregistered). Despite the fact that this appears to confirm one of the two main preregistered hypotheses of this study, we interpret the result cautiously for two reasons: First, as noted in the main body of the article, it is likely that participants influenced each other, undermining the statistical independence of the trials which is among the assumptions of the *z* test. Second, in the main study our results were in the opposite direction.

Combining the four classes described in this appendix with the three classes described in the main study, 104 discussion sections of roughly equal size were taught. Sufficient statistical power is thus available for a meaningful section-by-section analysis, avoiding the difficulty due to failure of independence and enabling an overview analysis of all of the available data. Pledge rates in the film discussion sections ranged from 5% to 81%. In the non-film sections, they ranged from 0% to 79%. We found no statistically detectable difference in mean pledge rates by condition (*M*_*film*_ = 40.7%, *M*_*nonfilm*_ = 37.5%, pooled *SD* = 19.5%, *t*(102) = 0.83, *p* = .41). Given this overall similarity, we interpret the data as not supporting our original hypothesis that exposure to the film would increase pledge rates.

Combining all seven classes also allows for a more powerful analysis of the influence of TA attitude on pledge rate. Students of TAs who were personally vegetarian appeared to pledge at slightly higher rates overall than students whose TAs were not personally vegetarian, though the effect size was small and near the cutoff of statistical detectability: 41% vs. 36% (304/742 vs. 400/1103, *z* = 2.03, *p* = .042, *φ* = .05). If we include only TAs who explicitly revealed whether they were vegetarian, the effect is larger and more statistically secure: 39% vs. 22% (205/522 vs. 64/289, *z* = 5.28, *p* < .001, *φ* = .17).
