# Supplementary material for: Students Eat Less Meat After Studying Meat Ethics
Source: Rev Philos Psychol. 2021 Nov 6;14(1):113–38. doi: 10.1007/s13164-021-00583-0 (PMC8571006; doi:10.1007/s13164-021-00583-0)
Supplement: Supplementary file 2 — (DOCX 20.7 kb) [file 13164_2021_583_MOESM2_ESM.docx]

**Appendix D, Post-Test**

The purpose of this experiment is to investigate students’ attitudes about several ethical issues. You will be asked 15 brief questions. The study should take about 5 minutes to complete.

---

You will receive [XXX extra credit] for participating in this study. If you choose not to participate, you may instead receive extra credit by writing two paragraphs on how the course has influenced your thinking on philosophical issues important to you and then submitting that assignment to your TA. You will receive extra credit either for completing this questionnaire or for writing two paragraphs, not for both.

---

This study is being conducted by Professor Schwitzgebel, who is a philosophy professor here at UCR, with the permission of your instructor for Philosophy [XXX], Professor [XXX]. Neither your TA nor Professor [XXX] will be told your answers to these questions. All identifying information will be stripped from your answers before Professor Schwitzgebel views the answers, so that no one will know how any particular student has answered. You will not be graded on your particular answers, and you should feel free to disagree with your professor and TA about the ethical issues at hand.

---

Participation is voluntary. You may discontinue participation at any time simply by closing your browser window.

---

If you have questions related to the study, please contact Professor Schwitzgebel at [contact info]. If you have questions about your rights or complaints as a research participant, please contact the IRB chairperson at [phone number] during business hours or contact them by email at [email].

---

By following the link below, you consent to participate in this study.

Some of the questions below may be similar to questions you received in a questionnaire earlier this term or in a previous term. It's fine if you answer the same way, and it's fine if you answer differently. Just express your opinion as it currently stands.

**Sexual intercourse outside of a committed, loving relationship is unethical. ***

- - **strongly agree**
  - **agree**
  - **slightly agree**
  - **neither agree nor disagree**
  - **slightly disagree**
  - **disagree**
  - **strongly disagree**

**I plan to avoid having sexual intercourse except in the context of a committed, loving relationship. ***

- - **strongly agree**
  - **agree**
  - **slightly agree**
  - **neither agree nor disagree**
  - **slightly disagree**
  - **disagree**
  - **strongly disagree**

**If I have sex outside of a committed, loving relationship, I should feel guilty about that. ***

- - **strongly agree**
  - **agree**
  - **slightly agree**
  - **neither agree nor disagree**
  - **slightly disagree**
  - **disagree**
  - **strongly disagree**

Some of the questions below may be similar to questions you received in a questionnaire earlier this term or in a previous term. It's fine if you answer the same way, and it's fine if you answer differently. Just express your opinion as it currently stands.

**Eating the meat of factory farmed animals is unethical. ***

- - **strongly agree**
  - **agree**
  - **slightly agree**
  - **neither agree nor disagree**
  - **slightly disagree**
  - **disagree**
  - **strongly disagree**

**For at least the next month, I will eat no factory farmed meat at all -- or if I find it too difficult to stick to that, I will eat it at most once per week. ***

- - **strongly agree**
  - **agree**
  - **slightly agree**
  - **neither agree nor disagree**
  - **slightly disagree**
  - **disagree**
  - **strongly disagree**

Top of Form

Bottom of Form

**If I eat factory farmed animals, I should feel guilty about that. ***

- - **strongly agree**
  - **agree**
  - **slightly agree**
  - **neither agree nor disagree**
  - **slightly disagree**
  - **disagree**
  - **strongly disagree**

Some of the questions below may be similar to questions you received in a questionnaire earlier this term or in a previous term. It's fine if you answer the same way, and it's fine if you answer differently. Just express your opinion as it currently stands.

**Spending large amounts of money on luxuries is unethical. Instead of spending large amounts of money on luxuries, people should give that money to charities that effectively aid people who suffer from poverty. ***

- - **strongly agree**
  - **agree**
  - **slightly agree**
  - **neither agree nor disagree**
  - **slightly disagree**
  - **disagree**
  - **strongly disagree**

**I plan to avoid spending large of amounts of money on luxuries. Money that I could spend on luxuries I instead plan mostly to donate to effective poverty-relief charities. ***

- - **strongly agree**
  - **agree**
  - **slightly agree**
  - **neither agree nor disagree**
  - **slightly disagree**
  - **disagree**
  - **strongly disagree**

Top of Form

Bottom of Form

**If I spend a large amount of money on luxuries, I should feel guilty about that. ***

- - **strongly agree**
  - **agree**
  - **slightly agree**
  - **neither agree nor disagree**
  - **slightly disagree**
  - **disagree**
  - **strongly disagree**

Some of the questions below may be similar to questions you received in a questionnaire earlier this term or in a previous term. It's fine if you answer the same way, and it's fine if you answer differently. Just express your opinion as it currently stands.

**Downloading music in violation of copyright laws is unethical. ***

- - **strongly agree**
  - **agree**
  - **slightly agree**
  - **neither agree nor disagree**
  - **slightly disagree**
  - **disagree**
  - **strongly disagree**

**I plan to avoid downloading music in violation of copyright laws. ***

- - **strongly agree**
  - **agree**
  - **slightly agree**
  - **neither agree nor disagree**
  - **slightly disagree**
  - **disagree**
  - **strongly disagree**

Top of Form

Bottom of Form

Top of Form

Bottom of Form

**If I download music in violation of copyright laws, I should feel guilty about that. ***

- - **strongly agree**
  - **agree**
  - **slightly agree**
  - **neither agree nor disagree**
  - **slightly disagree**
  - **disagree**
  - **strongly disagree**

**Did you watch the optional vegetarianism advocacy video “What Came Before?” (This video was shown at some section meetings but not at other section meetings.) ***

- - **no**
  - **I started watching but didn't finish**
  - **I watched the whole video**

**If you attended the discussion section meeting on vegetarianism, did you take the vegetarianism pledge at the end of the section meeting?**

- - **no**
  - **yes**
  - **did not attend section that day**
  - **prefer not to answer**Top of FormBottom of Form

Top of Form

Bottom of Form

**If you took the vegetarianism pledge at the end of the discussion section meeting, did you fulfill that pledge by not eating any factory farmed meat for the following 24 hours? (Any answer is okay, and neither the professor nor the TA will know how any individual student answered.) ***

- - **I did not take the pledge.**
  - **I took the pledge but I ended up eating some factory farmed meat during the next 24 hours anyway.**
  - **I took the pledge and I fulfilled the pledge by not eating any factory farmed meat during the next 24 hours.**
  - **I prefer not to answer.**

Thank you for completing our survey. Your answers will not be shared with your TA or professor and Prof. Schwitzgebel will remove all identifying information before viewing them. We will let your TA know that you have completed the survey so that you may receive your one half of one percentage point of extra credit. You may also print out this receipt page for your records if you wish.

---

If you have questions related to the study, please contact Professor Schwitzgebel at [contact info]. If you have questions about your rights or complaints as a research participant, please contact the IRB chairperson at [phone number] during business hours or contact them by email at [email].
