# Supplementary material for: Students Eat Less Meat After Studying Meat Ethics
Source: Rev Philos Psychol. 2021 Nov 6;14(1):113–38. doi: 10.1007/s13164-021-00583-0 (PMC8571006; doi:10.1007/s13164-021-00583-0)
Supplement: Supplementary file 3 — (DOCX 12.5 kb) [file 13164_2021_583_MOESM3_ESM.docx]

**PLEDGE FORM**

As part of an experiment by Professor Schwitzgebel (approved by UCR’s research ethics review board), we are interested in knowing how many students in various sections are willing to pledge not to eat the meat of any factory farmed animals for the next 24 hours. The purpose of this research is to examine the effects of philosophical teaching on students’ opinions and food choices. In several days, you will have the opportunity to complete a questionnaire expressing your opinions about various moral issues, including the issue of eating meat.

Pledging is voluntary and will have no influence on your grade in this course.

At no point will your TA or Professor Schwitzgebel know whether you have pledged, though you will later have an opportunity to report whether you have pledged, in a way that will not reveal your identity to the professor or TAs.

**By hand-drawing a figure in the space below, I am pledging not to eat the meat of any factory farmed animals for the next 24 hours.** (Please do not write your name or any other identifying information.)

**If you have pledged, please pick up a PLEDGE SHEET from the stack and take it home with you.**

If you have questions related to the study, please contact Professor Schwitzgebel at [contact info]. If you have questions about your rights or complaints as a research participant, please contact the IRB chairperson at [phone number] during business hours or contact them by email at [email].
