# Supplementary material for: Students Eat Less Meat After Studying Meat Ethics
Source: Rev Philos Psychol. 2021 Nov 6;14(1):113–38. doi: 10.1007/s13164-021-00583-0 (PMC8571006; doi:10.1007/s13164-021-00583-0)
Supplement: Supplementary file 4 — (DOCX 12.1 kb) [file 13164_2021_583_MOESM4_ESM.docx]

**PLEDGE SHEET**

If you have drawn a figure on the pledge form, please take **one copy** of this sheet. This will help us track how many students have pledged.

This sheet is for your use only. Do not return it to us. We request that you keep it with you during the 24 hours of your pledge, as a reminder of your pledge.

**I PLEDGE NOT TO EAT THE MEAT OF ANY FACTORY FARMED ANIMALS FOR THE NEXT 24 HOURS.**

If you have questions related to the study, please contact Professor Schwitzgebel at [contact info]. If you have questions about your rights or complaints as a research participant, please contact the IRB chairperson at [phone number] during business hours or contact them by email at [email].
